# Asthma Inflammatory Phenotypes: How Can We Distinguish Them?

**DOI:** 10.3390/jcm13020526

**Published:** 2024-01-17

**Authors:** Aleksandra Plavsic, Branka Bonaci-Nikolic, Branislava Milenkovic, Rada Miskovic, Natasa Kusic, Milan Dimitrijevic, Snezana Arandjelovic, Katarina Milosevic, Ivana Buha, Vesna Tomic Spiric

**Affiliations:** 1 Clinic for Allergy and Immunology, University Clinical Centre of Serbia, 11000 Belgrade, Serbia; branka.bonaci-nikolic@med.bg.ac.rs (B.B.N.); rada.miskovic@med.bg.ac.rs (R.M.);; 2 Faculty of Medicine, University of Belgrade, 11000 Belgrade, Serbia; branislava.milenkovic@med.bg.ac.rs (B.M.);; 3 Clinic for Pulmonology, University Clinical Centre of Serbia, 11000 Belgrade, Serbia; 4 Department of Pulmonology and Allergology, University Children’s Hospital, 11000 Belgrade, Serbia

**Keywords:** asthma, induced sputum, inflammatory phenotypes, biomarkers

## Abstract

Background and objectives: induced sputum is used to assess different inflammatory phenotypes in asthma, but is not used routinely. We aimed to determine the proportion of inflammatory asthma phenotypes based on induced sputum, to find biomarkers that can discriminate between phenotypes, and to evaluate biomarkers in patients with and without biological therapy in different inflammatory asthma phenotypes. Materials and Methods: this cross-sectional study investigated clinical characteristics, asthma control tests, skin prick test, impulse oscillometry (IOS), spirometry, induced sputum, biomarkers (IgE, eosinophils, fractional exhaled nitric oxide (FeNO), serum periostin, IL-5, IL-6, IL-8, IL-17A, IL-33) in 80 asthmatics. A total of 17/80 patients were treated with biologics (10 with omalizumab, 7 with benralizumab). Results: a total of 31% of patients had eosinophilic asthma (EA), 30% had mixed granulocytic asthma (MGA), 24% had paucigranulocytic asthma (PGA), and 15% had neutrophilic asthma (NA). The difference was found in blood eosinophils (*p* = 0.002), the highest observed in EA. The cut-off ≥ 240/μL eosinophils, with 64% sensitivity and 72.7% specificity, identified EA (AUC = 0.743, *p* = 0.001). A higher IL-8 level was associated with NA (*p* = 0.025). In 63 non-biologic asthma group, eosinophils were higher in EA than in NA, MGA, and PGA (*p* = 0.012, *p* = 0.028, and *p* = 0.049, respectively). A higher IL-17A was associated with EA without biologics (*p* = 0.004). A significantly higher IL-5 was found in EA treated with biologics, in comparison with EA without biologics (*p* = 0.043). The number of leucocytes and neutrophils was higher in MGA without biologics (*p* = 0.049, *p* = 0.019), while IL-5, IL-6, and IL-8 levels were higher in MGA treated with biologics (*p* = 0.012, *p* = 0.032, *p* = 0.038, respectively). Conclusions: EA and MGA were the most prevalent asthma phenotypes. Blood eosinophils can identify EA, both in patients with and without biologics. Apart from the clinical profile, a broad spectrum of biomarkers for assessing inflammatory phenotypes is necessary for an adequate therapy approach to patients with asthma.

## 1. Introduction

Asthma is characterized by airway inflammation, heterogeneous clinical presentation, different disease onset, variable therapy response, and unexpected exacerbation. The complexity of the pathophysiological mechanism and clinical picture are grouped under the diagnosis of asthma. It is a syndrome that consists of various endotypes and phenotypes. An endotype is defined by a molecular mechanism and therapy response, while a phenotype is an observable characteristic that results from the interaction between genotype and environment [1]. The concept of linking molecular characteristics to clinical features was introduced by Wenzel in 2012, and since then there has been ongoing research interest in asthma [2]. Many authors have evaluated the idea of a personalized approach based on endotypes and phenotypes in patients with asthma [3,4,5,6,7,8]. Stratification established on endotyping and phenotyping is important because it aims to find unique patient attributes and tailor the best therapy approach.

In clinical settings, it is not always easy to determine the inflammation and heterogeneity, two main asthma characteristics. To “measure” inflammation and to determine the type of inflammation, fiberoptic bronchial biopsies are the best method, but they are not routinely used and cannot be applied to a large asthmatic population. Induced sputum is a gold standard for the determination of inflammatory phenotypes. There are four different asthma phenotypes based on induced sputum analysis: eosinophilic, neutrophilic, mixed granulocytic, and paucigranulocytic [9]. There is no agreement about the cut-off values of neutrophils and eosinophils that should be used for the definition of asthma inflammatory phenotypes. In the literature, eosinophilic asthma is defined with the range of eosinophils in the sputum from >1.01% to ≥4%, and neutrophilic asthma is defined with a sputum neutrophil percentage from >40 to ≥76% [9,10,11,12]. The evaluation of inflammatory phenotypes in individual patients, besides giving information about inflammatory cell components, may guide therapeutic decisions and the need for corticosteroid therapy [13,14]. However, the induced sputum procedure is not applicable in everyday clinical settings, because it is time-consuming and requires highly trained personnel. So, finding adequate and easily available surrogate biomarkers that correspond to the type of airway inflammation is very important in clinical practice. Another important aspect of asthma evaluation is in achieving asthma control. There is no universal definition of asthma control, and it should include both the patient’s and the doctor’s perspectives, as well as objective parameters [15]. A combined approach to assessing asthma control from both symptom control and future risk of exacerbations is described in recommendations and is widely accepted [16,17].

Asthma is divided into Type 2 (T2) and Non-Type 2 asthma (non-T2). The characteristics of T2 asthma are an increased secretion of IL-4, IL-5, and IL-13 through Th2 and innate lymphoid cells type 2 (ILC2), increased IgE reactivity, elevated blood eosinophils and a fraction of exhaled nitric oxide (FeNO), a good response to inhaled corticosteroids (ICS), and biologics [14,17]. Non-T2 asthma is not well defined. It is a phenotype without T2 inflammation, without eosinophilia in serum and sputum, with more severe clinical presentation, and unresponsiveness to ICS, related to IL-6, IL-1b, IL-8, and IL-17A, but with no defined biomarkers [18,19]. Linking all features of different asthma phenotypes, including biomarkers with underlying inflammatory processes, seems to be essential in asthma and has been researched in many studies. Although biomarkers have been studied extensively, the data about inflammatory phenotypes based on induced sputum in clinical settings with heterogeneous asthma populations and larger sets of biomarkers are limited. The primary aim of our study was to determine the frequency of different inflammatory phenotypes (eosinophilic, neutrophilic, mixed granulocytic, and paucigranulocytic) in a cohort of patients with asthma at a specialized asthma department of a tertiary University Clinic, and to identify factors that could separate them. The secondary aim was to evaluate biomarkers in patients with and without biologics in different asthma phenotypes. 

## 2. Materials and Methods

This was a cross-sectional study that included 80 consecutive asthma outpatients, male and female, who were examined at the Clinic for Allergy and Immunology, University Clinical Centre of Serbia, Belgrade, from 2021 to 2023. The study was approved by the Ethics committee (1322/V-4) and written informed consent was obtained from all subjects. The inclusion criteria were ≥18 years of age, and an asthma diagnosis according to Global Initiative for asthma (GINA) [17]. The exclusion criteria were the use of systemic corticosteroid therapy, a history of viral, bacterial, and fungal respiratory tract infections in the past 4 weeks, and a documented COVID-19 infection less than 12 weeks before the study. Severe asthma was defined as asthma requiring high-dose ICS plus a second controller medication, and severe asthma exacerbation was defined as the occurrence of asthma-related hospitalization or visits to emergency care requiring the use of systemic corticosteroids for at least 3 days [20,21]. All patients were on maintenance asthma therapy. All participants underwent a detailed evaluation of clinical characteristics, personal and family history, triggers of exacerbations, medical asthma history, physical examination, asthma control assessment, skin prick test, FeNO measurement, impulse oscillometry (IOS), blood tests, spirometry, and induced sputum analysis on the same day, followed in this order.

Asthma control assessment was based on the following: asthma control test (ACT), Asthma Control Questionnaire (ACQ), and GINA asthma symptom control assessment [17,22,23]. Asthma was considered well controlled if an ACT score was over 20, partially controlled if an ACT score was 16–19 asthma, and uncontrolled if an ACT score was below 15 [22]. According to ACQ, a score of 0–0.75 points classifies patients as having well-controlled asthma, 0.76–1.5 as a ‘grey zone’, and >1.5 as having poorly controlled asthma [23]. Based on GINA asthma symptom control, asthma can be well controlled (none of the 4 answers is yes), partly controlled (1 or 2 answers is yes), and uncontrolled (3 to 4 answers is yes) [17]. The daily metered doses of ICS were classified based on GINA guidelines as low, medium, and high, based on equivalents of beclometasone dipropionate, budesonide, ciclesonide, fluticasone furoate, fluticasone propionate, mometasone furoate [17]. The skin prick test for common local inhalant allergens was performed according to the standard procedure and was considered positive if the papule diameter was >3 mm [24]. The complete blood count, total serum IgE (immunonephelometric, ARCHITECT), C-reactive protein (CRP) (immunoturbidimetric, ARCHITECT), and routine blood tests were collected. 

Serum levels of IL-5, IL-8, IL-17A, IL-33, and periostin were measured using ELISA (enzyme-linked immunosorbent assay) according to the manufacturer’s instructions. Briefly, standards and serum samples were incubated in wells precoated with a capture antibody highly specific for each cytokine. After washing the plate, biotinylated detection antibodies were added to each well, followed by streptavidin-HRP subsequent addition. After incubation and washing the plate, TMB substrate solution was added. After incubation in the dark and the addition of stop solution, the optical density (OD) of each well was read on a spectrophotometer using 450 nm as the primary wavelength, and 570–620 nm as the reference wave length. OD for each standard was plotted against a defined concentration, forming a standard curve. The standard curve was then used to accurately determine the concentration of cytokines in any sample tested. IL-5 levels were measured using PharmaGenie ELISA Kit (AssayGenie, Dublin, Ireland), while levels of IL-8, IL-17A, and IL-33 were quantified using LegendMax™ ELISA human kits (Biolegend, San Diego, CA, USA). Serum samples were analyzed without dilutions to determine IL-5, IL-8, IL-17A, and IL-33 levels, and their concentration is expressed in picograms per milliliter (pg/mL). Limit of detection for IL-5, IL-8, IL-17A, and IL-33 were 0.2, 0.3, 0.2, and 0.1 pg/mL, respectively. Periostin was measured by ELISA, according to the manufacturer’s instructions (Periostin, Biomedica Medizinprodukte GmbH, Wienna, Austria), using diluted samples (1 + 50). Concentrations of periostin are expressed in picomol per liter (pmmol/L). Serum IL-6 levels were obtained using the electrochemiluminescence method (ECLIA), according to the manufacturer’s instructions (Cobas, Elecsys IL-6, Roche, Basel, Switzerland), analyzed without dilutions, and concentrations of IL-6 are expressed in picograms per milliliter (pg/mL). 

The patients underwent a spirometry test (VyntusSPIRO, Jaeger, Bodnegg, Germany). FeNO measurement was performed at a flow of 50 mL/s (Medisoft, Dinant, Belgium), according to American Thoracic Society/European Respiratory Society (ATS/ERS) guidelines [25]. IOS was performed according to ERS recommendations, and small airway dysfunction (SAD) was defined as small airway resistance (R5 Hz–20 Hz, KPa/L/s) [26]. 

The spirometry test was performed before the induced sputum procedure and a forced expiratory volume in 1 s (FEV1) was recorded. The patients were given 200 μg of inhaled salbutamol, and post-bronchodilator FEV1 was measured after 10 min. Then, a 4.5% saline inhalation via ultrasonic automizer was given at 5 min intervals, for a total time of 20 min. If there was a fall in FEV1 of 20% from baseline or symptom appearance, the test was stopped and discontinued. The induced sputum procedure was performed using the method defined by Djukanovic et al. [27]. The whole sputum sample was sent for examination. The specimen was treated with 0.1% dithiothreitol, and then was filtered and centrifugated. The fixation and staining followed using May Grunwald Giemsa. The cell count was determined by counting 500 non-squamous cells and is reported as the relative numbers of eosinophils, neutrophils, macrophages, lymphocytes, and bronchial epithelial cells, expressed as a percentage of total non-squamous cells. The patients were divided into 4 inflammatory phenotypes, according to the sputum cells analyses: eosinophilic asthma (EA) was defined as ≥3% eosinophils and <61% neutrophils; neutrophilic asthma (NA) was defined as <3% eosinophils and ≥61% neutrophils; mixed granulocytic asthma (MGA) as ≥3% eosonophils and ≥61% neutrophils; and paucigranulocytic asthma (PGA) was defined as <3 eosinophils and <61% neutrophils [28]. 

### Statistical Analysis

Categorical data are presented as absolute and relative numbers in percentages. Numerical data are described by the arithmetic mean with standard deviation, or median with a range from minimum to maximum, depending on the data distribution. Normal distribution was evaluated using mathematical (Shapiro–Wilk test) and graphical (histogram, box-plot) methods. Four independent subgroups (EA, NA, MGA, and PGA) were compared, according to categorical data by chi-square test or Fisher’s exact test if the criteria for the previously mentioned one were not met. Also, they were compared according to numerical data by one-way ANOVA with Tuckey post hoc testing or Kruskal–Wallis with Mann–Whitney as a post hoc testing method, depending on the data distribution. To explore factors that can distinguish different asthma phenotypes, we applied logistic regression analysis (Backward: Wald method). First, univariate, and then multivariate analysis was performed. All significant factors from univariate analyses went into multivariate models. Diagnostic performances of eosinophils count as a potential biomarker for EA phenotype, and were evaluated by ROC curve, sensitivity, and specificity. Cut-off value was chosen for the point where the Youden’s index had the highest value. All statistical methods were considered significant if *p* value was ≤0.05. Complete statistical analysis was performed in IBM Corp., released 2012. IBM SPSS Statistics for Windows (Version 21.0. IBM Corp., Armonk, NY, USA).

## 3. Results

This cross-sectional study included a total of 80 patients with asthma with an average age of 43.65 ± 12.72 years and a male-to-female ratio of 1:2. EA asthma was present in 25 patients (31%), 12 had NA (15%), 24 patients had MGA (30%), and 19 PGA (24%), according to induced sputum analysis. Socio-demographic, functional, and clinical characteristics of all patients with asthma, according to four inflammatory phenotypes, are presented in Table 1. There was an equal distribution of smokers, ex-smokers, and non-smokers (*p* = 0.960), with an average smoking duration of 14 years. The median asthma duration was 7 years. The most common comorbidity was allergic rhinitis, in 79% of patients. Almost half had a positive family history of allergic rhinitis and 39% for asthma. 

Small airway resistance, as measured by IOS, was detected in 45% of the whole cohort, with a prevalence of 56% in EA, 45.8% in MGA, 42.1% in PGA, and 25% in NA, but with no differences among phenotypes (Table 1). There was a significant difference in mean expiratory flow at 75% of vital capacity (MEF75) between four inflammatory asthma phenotypes (*p* = 0.042) (Table 1). It was the highest in MGA. It was significantly higher in MGA than in NA, and also in MGA than in PGA (*p* = 0.025 and *p* = 0.019, respectively). There was no difference in MEF75 between other asthma phenotypes (EA vs. NA *p* = 0.455, EA vs. MGA *p* = 0.062, and EA vs. PGA *p* = 0.297).

Uncontrolled disease was recorded in 41.3% of patients, according to GINA; the average ACT score was 16.5, suggesting partially controlled asthma, and the average ACQ was 1.5 (Table 2). Regarding therapy, the most prevalent drugs were antihistamines (72.5%), nasal glucocorticoids (58.8%), and leukotriene receptor antagonist (LTRA) (57.5%), followed by asthma therapy: short-acting beta 2 agonist (SABA) in 41.3%, 37.5% were taking inhaled corticosteroid/long-acting beta 2 agonists (ICS/LABA) maintenance-and-reliever-therapy with ICS-formoterol (MART), and 36.3% ICS/LABA (Table 2). The biologics were administered to 17 patients; 10 were on omalizumab and 7 were on benralizumab. There was no difference in any of the evaluated socio-demographic and clinical characteristics, disease control, and therapy between the four inflammatory phenotypes of asthma. 

**Figure 1:**
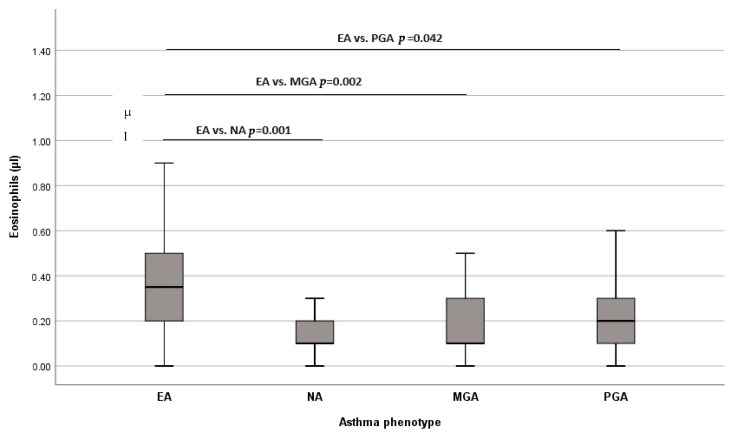
Eosinophils (μL) in different asthma phenotypes. Abbreviations: EA: eosinophilic asthma; NA: neutrophilic asthma; MGA: mixed-granulocytic asthma; PGA: paucigranulocytic asthma.

Biomarkers in all patients with asthma and according to phenotypes were evaluated. The mean IgE was 121.5 IU/mL (56.2–364.5 IU/mL) and FeNO 23.5 ppb (12.1–37.7 ppb) (Supplementary Table S1). There was a significant difference in the number of blood eosinophils between four inflammatory asthma phenotypes (*p* = 0.002) (Figure 1). The level of eosinophils was significantly higher in EA than in NA (*p* = 0.001), MGA (*p* = 0.002), and PGA (*p* = 0.042), while there was no difference between NA and MGA (*p* = 0.376), NA and PGA (*p* = 0.114), MGA, and PGA (*p* = 0.402). There was no difference in IgE, FeNO, periostin, IL-5, IL-6, IL-8, IL-33, and IL-17A serum levels among phenotypes (Supplementary Table S1).

Using logistic regression, we analyzed factors associated with different asthma phenotypes (Table 3). Factors that were independently associated with EA were smoke as a trigger (OR = 5.966, 95%CI OR = 1.53–23.30, *p* = 0.010), lower MEF25 (OR = 0.964, 95%CI OR = 0.94–0.99, *p* = 0.019), and higher CRP (OR = 1.246, 95%CI OR = 1.01–1.54, *p* = 0.043) (Table 3). A higher level of IL-8 was the only factor independently associated with NA phenotype (OR = 1.009, 95%CI OR = 1.01–1.02, *p* = 0.025). The only factor that was independently associated with MGA was higher MEF50 (OR = 1.030, 95%CI OR = 1.01–1.06, *p* = 0.023) (Table 3). The only factor possibly associated with PGA asthma phenotype was the nasal glucocorticoids non-use in therapy.

**Figure 2:**
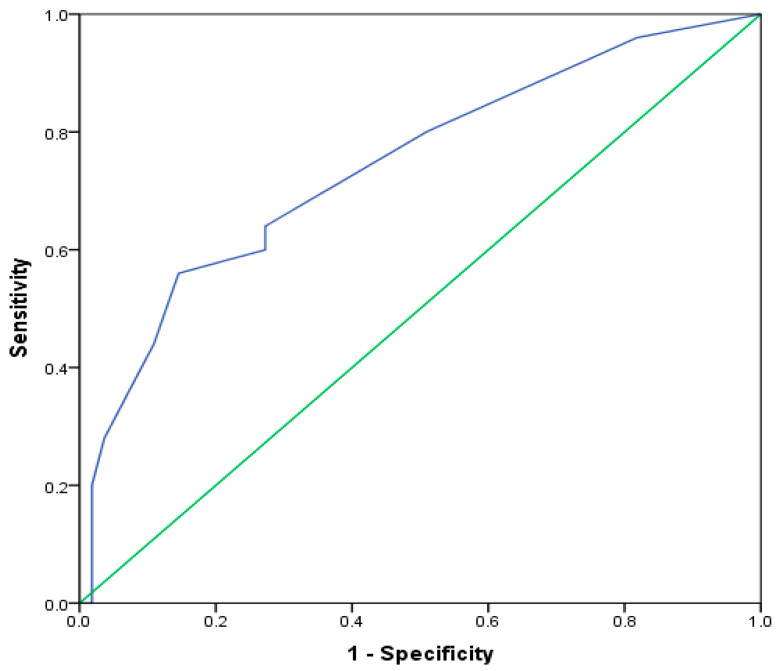
ROC curve for eosinophils counts as a diagnostic marker for EA phenotype (cut-off value ≥ 240/μL, Sn = 64.0%, and Sp = 72.7%). Abbreviations: EA: eosinophilic asthma.

We investigated the possibility of using an eosinophils count to identify patients with the EA phenotype, using ROC analysis. We found that the cut-off value of eosinophils ≥240/μL, with 64% sensitivity and 72.7% specificity (area under the ROC curve = 74.3% *p* = 0.001), distinguished patients with EA phenotypes (Figure 2).

We analyzed the biomarkers in 63 patients who were not taking biologics (non-biologic group) (Table 4). There was a significant difference in the number of eosinophils and the level of IL-17 between four inflammatory asthma phenotypes (*p* = 0.034 and *p* = 0.004). Both biomarkers were the highest in EA phenotype. A number of eosinophils was significantly higher in EA than in NA, MGA, and PGA phenotypes (*p* = 0.012, *p* = 0.028, and *p* = 0.049, respectively), while IL-17 was significantly higher in EA than in NA and PGA (*p* = 0.016 and *p* = 0.002, respectively), and in MGA than in PGA phenotype (*p* = 0.042). 

**Figure 3:**
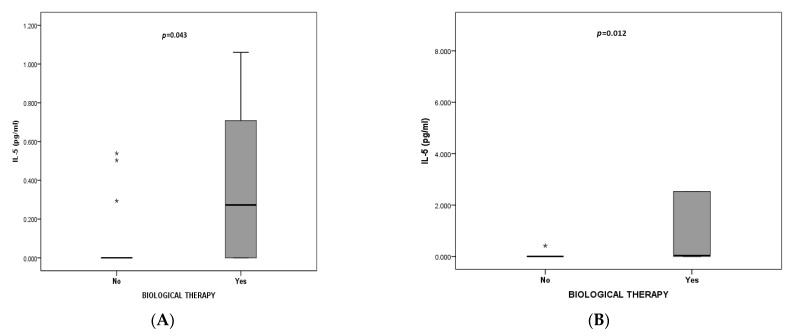

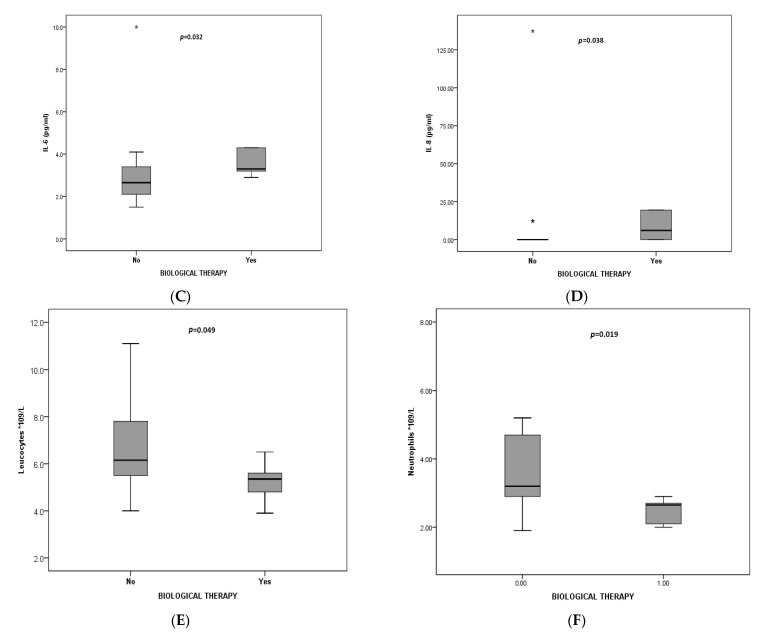
Biomarkers in biologic and non-biologic group according to asthma phenotypes. (**A**) IL-5 in EA biologic and EA non-biologic group (*p* = 0.043) (**B**) IL-5 in MGA biologic and MGA non-biologic group (*p* = 0.012); (**C**) IL-6 in MGA biologic and MGA non-biologic group (*p* = 0.032); (**D**) IL-8 in MGA biologic and MGA non-biologic group (*p* = 0.038); (**E**) Leucocytes in MGA biologic and MGA non-biologic group (*p* = 0.049); (**F**) neutrophils in MGA biologic and MGA non-biologic group (*p* = 0.019). Abbreviations: EA: eosinophilic asthma, MGA: mixed-granulocytic asthma. * Data are presented by box-plots where the central line represents median, lower, and upper edge of the box Q1 and Q3, and whiskers minimum and maximum values, *p* values according to Mann–Whitney test are presented.

Finally, we decided to divide patients into those who were in the biologic group (*n* = 17) and those in the non-biologic group (*n* = 63), and to evaluate biomarkers according to phenotypes. There was a significant difference in IL-5 between the EA biologic and the EA non-biologic groups (*p* = 0.043) (Figure 3A). IL-5 was higher in those on biological medications. A number of leucocytes and neutrophils, and IL-5, IL-6, and IL-8, differed between MGA patients who were and were not treated with biological therapy (*p* = 0.049, *p* = 0.019, *p* = 0.012, *p* = 0.032, and *p* = 0.038, respectively). IL-5, IL-6, and IL-8 were higher in the MGA biologic group, while leucocytes and neutrophils were higher in the MGA non-biologic group (Figure 3B–F) (Supplementary Table S2).

## 4. Discussion

The identification of asthma phenotypes is the goal of asthma patient evaluation because it provides information about the prominent asthma characteristic inflammation. Induced sputum is the best option for the determination of inflammatory phenotypes. However, it is not always an accessible method, and there are no recommendations on when and how often it should be used. In our study, we aimed to evaluate the proportion and characteristics of phenotypes in a cohort of patients with asthma using induced sputum. Our cohort consisted of patients with different levels of asthma control, inhalation therapy, and biologics, and an almost equal distribution of smokers, ex-smokers, and non-smokers. It has been shown that current smokers have reduced sputum eosinophils compared with never-smokers, and ex-smokers have more sputum neutrophils and a similar proportion of sputum eosinophils compared to never-smokers [29]. In our cohort, we did not find a difference in the distribution of smoking habits within four inflammatory asthma phenotypes (*p* = 0.960). The most prevalent comorbidity was allergic rhinitis, present in 78.8%. This may be explained by the profile of patients in our hospital. We are a referent tertiary center for allergy and immunology, and patients with suspected allergic rhinitis are referred to our hospital. The mean IgE of 121.5 IU/mL (56.2–364.5 IU/mL), the most frequently reported clinical manifestation (nasal congestion), triggers asthma exacerbation (allergens), and a high prevalence of positive family history for asthma and allergic rhinitis can be also attributed to high allergic rhinitis prevalence. Allergic rhinitis could have affected lung function in our cohort, considering the most frequently applied therapies were antihistamines and nasal corticosteroids.

There was an almost equal distribution of EA (*n* = 25) and MGA (*n* = 24), while 19 subjects had PGA and 12 subjects had NA in the whole cohort. Other studies have also found EA to be the most prevalent phenotype, presented in 41%, 40%, and 46.9% of patients, respectively, but the distribution of the other three phenotypes was varying [9,30,31]. The predominance of the PGA phenotype was seen among 176 patients with asthma in China, in 42.6% [32]. The differences in predominant phenotype may be explained by the diversity of cohorts. In other cross-sectional studies, like ours, the patients were also evaluated for maintenance therapy that could affect the “initial” phenotype, but a more severe asthma population was included [30,31,32]. 

For the whole cohort, there was a significant difference in eosinophils’ blood numbers, the highest recorded in EA (*p* = 0.002). The level of eosinophils was significantly higher in EA when compared to other phenotypes: NA (*p* = 0.001), MGA (*p* = 0.002), and to PGA (*p* = 0.042). The value of ≥240/μL blood eosinophils could identify EA, with 64% sensitivity and 72.7% specificity (AUC = 0.74, *p* = 0.001). We did not confirm the correlation between the number of eosinophils in blood and the % of eosinophils in sputum among patients with EA (ρ = −0.03, *p* = 0.863). The correlation between serum and sputum eosinophilia is seen in many studies [31,33,34]. Schleich et al. have shown this connection in more than 500 asthmatics, and they have set the cut-off value of 220/μL of blood eosinophils to identify ≥3% eosinophils in sputum [35]. The cut-off for eosinophils of 270/μL and 300/μL to identify sputum eosinophilia is determined in other studies [34,36]. However, there are no recommended blood eosinophils values that may be used as a surrogate marker for sputum eosinophilia. Also, no unique cut-off is established for identifying sputum eosinophilia. Simpson has shown that the cut-off value of 3% sputum eosinophils is better reproducible as a discriminator of airway eosinophilia than 2% [28]. This cut-off has been used in many studies based on these findings. Some authors have questioned the use of blood eosinophils as a single marker for detecting sputum eosinophilia [37,38]. Como et Bel concluded that eosinophilia and FeNO are diagnostic biomarkers to rule in or to rule out EA, and they have suggested a 400/μL blood eosinophils value for identifying EA [39]. In a comprehensive literature review, the following numbers of eosinophils were assessed: >150/μL in severe eosinophilic asthma, up to >400/μL in poorly controlled with high-dose ICS, >200/μL in unselected patients, and >270/μL and >300/μL in mild to severe asthma [40]. These numbers corresponded to different sputum eosinophils’ cut-off values: >2%, ≥2%, ≥2.5%, >3%, and ≥3%, respectively. The conclusion was that blood eosinophils are an appropriate biomarker for airway eosinophilia, but are not completely adequate for phenotype discrimination, even in combination with other biomarkers, especially in severe asthma.

Our study did not confirm the correlation between FeNO and inflammatory phenotypes. This was also reported by other authors [37,38,41]. On the contrary, the connection between FeNO and sputum eosinophilia was found in other studies [33,35]. The recommended value >50 ppb is established by ATS for identifying EA and <25 ppb for ruling it out [42]. In our cohort, the mean FeNO was 23.5 ppb (12.1–37.7 ppb), with no differences among phenotypes. Our cohort was comprised of patients with mainly allergic rhinitis, on maintenance therapy, and more than half of them were smokers. All these factors (allergy, therapy, smoking) could have contributed to the FeNO value. Some authors propose multiple biomarkers, such as high FeNO and eosinophils, to improve the identification of the sputum EA phenotype [39]. However, the combination of eosinophils, FeNO, and IgE was not found to be beneficial in sputum eosinophilia determination [34,40]. 

The IOS parameters, using an interpretation of R5–R20 Hz, are considered good markers of SAD [43]. We found a high prevalence of small airway resistance in EA of 56%, and 45.8% in MGA, 42.1% in PGA, and 25% in NA, but no difference was seen in SAD among phenotypes. A significant difference in MEF75 between four inflammatory asthma phenotypes was found (*p* = 0.042), the highest observed in MGA. The role of MEF 25, MEF50, MEF75, and MEF25–75, the mid-maximal expiratory flow rate (MMEF), has been established as a marker of SAD in asthma. [44,45]. A prospective, multicenter study has found that SAD was present in all severity asthma groups according to GINA, and was highly prevalent in severe disease [46]. SAD is associated with poor asthma control and more exacerbations [47]. Our findings of 45% SAD in the whole cohort, as well as a discrepancy in MEF75 among different phenotypes, suggest that SAD evaluation may be an important part of inflammatory phenotypes assessment. Not so many studies have looked at the connection between SAD and inflammatory phenotypes. The study of Abdo et al. has addressed this issue [48]. The SAD in 197 patients with asthma, divided into four phenotypes based on induced sputum, was followed for one year. The EA and MGA patients had worse SAD features compared to the other two phenotypes. Patients with persistent elevated eosinophils and neutrophils had worse SAD after follow-ups. The change in sputum eosinophils was an independent risk factor of lung function change and a connection between SAD and airway eosinophilia was shown. Our results also showed that small airway resistance, as a marker of SAD, was the most prevalent in EA and MGA, but we found MEF75 to be the highest in MGA. Our results suggest a careful interpretation of spirometry when evaluating SAD. MEF75 is only one of the spirometry parameters, part of MMEF. There is no recommendation of which spirometry parameter is the best predictor of SAD [49]. The study that compared these two methods found that for normal lung function IOS may be more sensitive, while spirometry is a more sensitive method for detecting SAD in abnormal lung function [50].

We analyzed the characteristics that could discriminate phenotypes. EA was defined by a greater number of severe exacerbations during the previous year, smoke as a trigger, nasal glucocorticoids in therapy, smaller MEF25 and MEF50, as well as greater CRP and periostin blood levels. Factors that were independently associated with EA were smoke as a trigger (OR = 5.966, 95%CI OR = 1.53–23.30, *p* = 0.010), lower MEF 25 (OR = 0.964, 95%CI OR = 0.94–0.99, *p* = 0.019), and higher CRP. Other studies have also found that EA is associated with poor control and higher periostin levels [35,51,52,53]. However, the connection between serum periostin and sputum eosinophils was not detected by other researchers [34]. Our EA cohort was composed of patients with AR, so nasal glucocorticoid use was expected. Higher CRP was an independent factor associated with EA. Highly sensitive CRP was found to be higher in 45 patients with asthma compared to healthy subjects, and significantly correlated with eosinophils and neutrophils in the sputum [54]. A recent study also found that EA was associated with higher CRP, among other inflammatory markers, and higher periostin compared to NA [55]. CRP is a general inflammatory marker that may be elevated in many diseases and infections. Our results may indicate that EA patients have a higher level of inflammation that is ongoing and persistent and, as such, can be assessed through different biomarkers of inflammation, such as CRP. However, this result should be interpreted carefully considering CRP sensitivity. Regarding NA, we have found that IL-8 was an independent factor associated with this phenotype. This result was also seen in a study conducted in China, but besides IL-8 in blood, it was found that sputum IL-8 and serum IL-17 could be also predictors of NA [32]. Higher IL-8 was recorded in NA, but also in MGA [30]. Sputum IL-8 and neutrophil elastase protein, IL-8RA, and IL-8RB gene expression were increased in NA [56]. It is proposed that after epithelial cells and alveolar macrophage activation, proinflammatory cytokines (IL-6, IL-1b, IL-8) are secreted, causing the chemoattraction of neutrophils and neutrophil activation [57]. The IL-17 pathway in NA was also found to be important, but we did not find an association between IL-17A and NA compared to other studies [32,58,59]. The NA is a complex phenotype, with incompletely understood pathophysiological mechanisms. The MGA phenotype was characterized by the presence of allergic rhinitis in the family, physical activity as a trigger, allergic immunotherapy, and better lung function. The only factor that was independently associated with MGA was higher MEF50. Our results of allergic features of these phenotypes indicate “the mix” of allergic and non-allergic factors expressed in this subgroup. However, better lung function was not observed in other studies [35,38,48]. Of all phenotypes, PGA was studied the least. It is a phenotype with no prominent inflammation, with good lung function and a good response to therapy [30,60]. Our results also pointed out the unspecific profile of this phenotype and mild clinical presentation. 

We did not find any difference in other serum biomarkers (IgE, periostin, IL-5, IL-6, IL-8, IL-17A, and IL-33) among the four inflammatory phenotypes. These immunological markers may correspond to pathophysiological characteristics of asthma: periostin, IgE, IL-5, and IL-33 for T2 asthma; IL-6, IL-8, IL-17A, and IL-33 for non-T2 asthma. Obtaining cytokines from induced sputum, BAL or biopsy provides better local cytokine information about inflammation, but serum samples may give information about systemic inflammation [61]. The interplay between different subsets of Th1, Th2, Th17, and ILC-2, the overlap and change in one dominant immunological response over the other, depending on exposure to different factors and cytokine profile surrounding makes the pathogenesis of asthma still not completely understood [62]. Our results that found no discrepancy among prominent biomarkers of different pathophysiological pathways may support this plasticity and the overlap of various immunological interactions in asthma, caught at one point in time, as we have performed a one-time analysis of induced sputum. 

Biological therapy is effective in improving ACT and exacerbations, as well as FeNO [63]. Also, biologicals can reduce the number of blood eosinophils in both responders and non-responders of EA, and they cannot be used as a predictive marker of EA in those patients [64]. We decided to evaluate biomarkers in 63 patients who were not taking biologics. Similar phenotype distribution, as in the whole cohort, was seen: 18 had EA (29%), 10 NA (16%), 18 MGA (29%), and 17 PGA (27%), confirming the observation that EA and MGA are the most prevalent. Also, the number of eosinophils was significantly higher in EA than in NA, MGA, and PGA phenotypes (*p* = 0.012, *p* = 0.028, and *p* = 0.049, respectively), confirming the results obtained from the whole cohort. IL-17 A was significantly higher in EA than in NA and PGA (*p* = 0.016 and *p* = 0.002, respectively) and in MGA than in PGA phenotype (*p* = 0.042). IL-17 is linked to NA, but our results have found that EA without biologics had the highest IL-17A levels. These findings may be explained by the mixed Th2/Th17 endotype that could be dominant in our EA non-biological group, as Th2/Th17 cell subsets are discovered [65,66]. Also, animal models support the idea that IL-17 A effects Th2 cell-mediated eosinophilic airway inflammation [67]. It was found that IL-17 is associated with uncontrolled asthma compared to controlled patients with asthma, regardless of atopic status, and combined with upregulated Th2 cytokines, may point to refractory asthma [68]. Also, a dual Th2/Th17 subset can lead to inflammatory cell recruitment and asthma exacerbations [69].

Finally, we wanted to compare biomarkers in two groups of patients: 63 in the non-biologic group and 17 in the biologic group, according to four phenotypes. There was a significant difference in IL-5 between the EA biologic and EA non-biologic groups (*p* = 0.043), with higher IL-5 in those who were taking biologics. It has been shown that the administration of benralizumab and mepolizumab causes an increase in serum IL-5 [70,71,72]. In our EA biologics group, six patients out of seven were receiving omalizumab, and one was receiving benralizumab. It was found that omalizumab decreases IL-5 levels [73]. Our results may suggest that omalizumab patients also had an IL-5 pathway endotype, so higher serum IL-5 may indicate ongoing, persistent eosinophilic inflammation that was not targeted with biologics, and switching to other biologics can be considered. We found that IL-5, IL-6, and IL-8 were higher in the MGA biologic group (*p* = 0.012, *p* = 0.032, and *p* = 0.038, respectively), while leucocytes and neutrophils were higher in the MGA non-biologics group (*p* = 0.049, *p* = 0.019). A high IL-5 may be the result of benralizumab therapy that was given to four out of six patients in the MGA biologic group. The higher IL-8 suggests a more prominent neutrophilic part in the mix inflammation of MGA, and no effects of biologic therapy regarding neutrophil inflammation. IL-8 was found to be elevated in MGA in another study [30]. A higher IL-6 may be due to persistent systemic inflammation in the MGA biologic group. Other authors report NA to be associated with inflammatory systemic markers CRP and IL-6 [56,60].

The determination of airway inflammation is of great importance in asthma. How do we identify inflammatory phenotypes in everyday practice? How many inflammatory phenotypes exist? Considering the complexity of asthma pathogenesis, can we assume that phenotypes overlap and change over the course of disease in some patients, or is there a great heterogeneity within one phenotype? How many patients have stable phenotypes? A recent study showed that 30 out of 68 patients with severe eosinophilic asthma had switched from mepolizumab to benralizumab, after a median of 21 months, suggesting the change in the immunological background of patients who responded well in the beginning [74]. The authors hypothesized the possibility of an IL-5-independent eosinophilic inflammation. Since the stability of an eosinophilic phenotype based on induced sputum measurement was confirmed in some studies, the significance of a single induced sputum measurement, as a marker of airway inflammation, may be considered in everyday practice [75,76]. Other studies did not confirm the sputum stability [77]. It has been suggested that there is only one eosinophilic phenotype, and the other three phenotypes may represent the therapeutic success or failure of eosinophilic asthma. Some authors argue that PGA is not a “true” phenotype, but a well-treated group of patients [30]. There are also doubts about NA being a distinct phenotype, considering the ICS effect on inflammation [13,66]. 

Our study has some limitations. Considering the study design, we have no information about the initial inflammatory phenotype at the time of asthma diagnosis, so we cannot include the impact of asthma therapy and other factors on current inflammation. The data was collected in a single center that specializes in allergy conditions with a small sample size. We believe that the use of IOS as an additional tool for characterizing inflammatory phenotypes, as well as broader cytokine analysis and comparison of biomarkers in phenotypes based on biologic use presents the study’s strength.

It has been shown that induced sputum is the best method for eosinophilic evaluation, compared to non-invasive-based algorithms [78]. In an ideal setting, when evaluating an asthma patient for the first time, before initializing therapy, we believe that induced sputum with IOS, alongside “standard testing” (spirometry, FeNO, IgE, blood eosinophils), and clinical profiles should be incorporated in phenotyping. However, the question of the frequency of performing induced sputum and the accessibility of IOS and induced sputum in clinical practice still remains.

## 5. Conclusions

In our cohort of patients with asthma, the dominant phenotypes were EA and MGA. Blood eosinophilia is a marker that distinguishes EA from other phenotypes, both in patients with and without biologics. IL-17A could be included as a marker for EA phenotype in patients without biologics. Higher neutrophils and leucocytes are associated with MGA patients without biological therapy. Higher serum IL-5 can be considered as a marker of EA for patients on biologics, while higher serum IL-5, IL-6, and IL-8 can be used as biomarkers for MGA patients treated with biologics. Except for induced sputum, different biomarkers may be an important tool for assessing asthma inflammatory phenotypes.

## Figures and Tables

**Table 1:** Socio-demographic, clinical, and functional characteristics of patients with asthma.

Characteristic	Total	EA *n* = 25	NA *n* = 12	MGA *n* = 24	PGA *n* = 19	*p* *
Age (years), mean ± SD	43.65 ± 12.72	45.44 ± 13.62	41.67 ± 12.46	41.71 ± 13.78	45.00 ± 10.56	0.677
Male	23 (28.8)	8 (32.0)	3 (25.0)	7 (29.2)	5 (26.3)	0.966
Female	57 (71.3)	17 (68.0)	9 (75.0)	17 (70.8)	14 (73.7)	
Smoking habit, *n* (%)						
Active smoker	20 (25.0)	6 (24.0)	2 (16.7)	6 (25.0)	6 (31.6)	0.960
Ex-smoker	15 (18.8)	4 (16.0)	2 (16.7)	5 (20.8)	4 (21.1)	
Non-smoker	55 (56.3)	15 (60.0)	8 (66.7)	13 (54.2)	9 (47.4)	
Smoking duration (years), med (min–max)	14 (1–50)	15 (3–27)	15 (10–20)	10 (5–50)	20 (1–30)	0.817
Asthma duration (years), med (min–max)	7 (0–52)	10 (1–45)	3.5 (0–23)	6.5 (0–50)	7 (1–52)	0.155
BMI, mean ± SD	26.43 ± 4.64	26.52 ± 2.80	24.67 ± 4.46	27.26 ± 6.24	26.39 ± 4.36	0.483
Number of severe exacerbations during last year, med (min–max)	0 (0–10)	1 (0–8)	0 (0–7)	0 (0–4)	0 (0–10)	0.134
Allergic rhinitis, *n* (%)	63 (78.8%)	18 (72.2)	10 (83.3)	20 (83.3)	15 (78.9)	0.769
Nonallergic rhinitis, *n* (%)	15 (18.8)	6 (16.7)	42(16.7)	4 (16.7)	3 (15.8)	0.882
Drug allergy, *n* (%)	22 (27.5)	6 (24.0)	4 (33.3)	7 (29.2)	5 (26.3)	0.939
CRSwNP, *n* (%)	12 (15.0)	5 (20.0)	2 (16.7)	3 (12.5)	2 (10.5)	N/A
CRSsNP, *n* (%)	10 (12.5)	2 (8.0)	1 (8.3)	3 (12.5)	4 (21.1)	N/A
GERB, *n* (%)	10 (12.5)	4 (16.0)	0 (0.0)	3 (12.5)	3 (15.8)	N/A
Allergic rhinitis, family, *n* (%)	39 (48.8)	9 (36.0)	4 (33.3)	16 (66.7)	10 (52.6)	0.113
Asthma, family, *n* (%)	31 (38.8)	9 (36.0)	6 (50.0)	9 (37.5)	7 (36.8)	0.858
FEV1, % predicted, mean ± SD	92.60 ± 14.54	90.56 ± 17.09	91.42 ± 14.58	95.86 ± 11.98	91.92 ± 14.30	0.619
FEV1/FVC %, mean ± SD	71.36 ± 8.54	69.78 ± 6.45	71.46 ± 9.25	74.59 ± 10.68	69.28 ± 6.67	0.142
MMEF, %, mean ± SD	67.15 ± 21.55	60.20 ± 21.89	67.92 ± 22.02	74.71 ± 21.10	66.26 ± 19.72	0.170
MEF25, %, med (IQR)	50.0 (37.2–62.7)	45.0 (30.9–55.5)	48.0 (40.5–72.22)	52.5 (36.0–63.0)	56.0 (38.0–68.0)	0.230
MEF50, %, med (IQR)	70 (56.0–87.7)	68.0 (37.4–77)	69.0 (53.5–69.0)	79.0 (61.0–95.0)	74.7 (53.0–82.0)	0.185
MEF75, %, med (IQR)	93.0 (76.2–109.0)	98.0 (55.8–109.0)	87.5 (73.5–97.7)	105.0 (81.7–111)	86.9 (71.0–99.0	0.042
SAD, *n* (%)	36 (45.0)	14 (56.0)	3 (25.0)	11 (45.8)	8 (42.1)	0.357
COVID 19 infection, *n* (%)	62 (78.5)	18 (75.0)	9 (75.0)	17 (70.8)	18 (94.7)	0.255

* For the level of significance of 0.05, according to one-way ANOVA, chi-square test or Kruskal–Wallis test. Abbreviations: EA: eosinophilic asthma; NA: neutrophilic asthma; MGA: mixed-granulocytic asthma; PGA: paucigranulocytic asthma; BMI: body mass index; CRSwNP: chronic rhinosinusitis with nasal polyp, CRSsNP: chronic rhinosinusitis without nasal polyp, GERB: gastro-esophageal reflux disease; FEV1: forced expiratory volume in 1 s; FVC: forced vital capacity; MMEF: mid–maximal expiratory flow rate; MEF25 maximal expiratory flow at 25% of vital capacity; MEF50 maximal expiratory flow at 50% of vital capacity; MEF75 mean expiratory flow at 75% of vital capacity, SAD: small airway dysfunction; N/A: not applicable.

**Table 2:** Asthma control assessment and asthma medical history.

Characteristic	Total	EA *n* = 25	NA *n* = 12	MGA *n* = 24	PGA *n* = 19	*p* *
GINA symptom control, *n* (%)						
Well-controlled	17(21.3)	4 (16)	2 (16.7)	7 (29.2)	4 (21.1)	
Partly controlled	30 (37.5)	10 (40)	4 (33.3)	8(33.4)	8(42.1)	0.928
Uncontrolled	33 (41.3)	11 (44)	6 (50)	9 (37.5)	7 (36.7)	
ACT, med (IQR)	16.5 (12.0–22.0)	16.0 (11.0–20.5)	16.5 (12.2–24.7)	18.5 (11.5–24.7)	16.0 (12.0–21.0)	0.572
ACQ, med (IQR)	1.5 (0.5–2.6)	2.0 (0.7–3.0)	1.2 (0.3–2.4)	1.2 (0.4–3.0)	1.7 (0.8–2.5)	0.581
Therapy						
Antihistamine, *n* (%)	58 (72.5)	21 (84)	9 (75)	17 (70.8)	11 (57.9)	0.288
Nasal glucocorticoids, *n* (%)	47 (58.8)	19 (76)	6 (50.0)	15 (62.5)	7 (36.8)	0.062
Allergic immunotherapy	12 (15)	3 (12)	0 (0)	7 (29.2)	2 (10.5)	N/A
ICS, *n* (%)	10 (12.5)	4 (16)	1 (8.3)	4 (16.7)	1 (5.3)	N/A
SABA *n* (%),	33 (41.3)	8 (32)	6 (50)	11 (45.8)	8 (42.1)	0.688
LAMA, *n* (%)	9 (11.3)	3 (12)	1 (8.3)	4 (16.7)	1 (5.3)	N/A
LTRA, *n* (%)	46 (57.5)	18 (72.0)	7 (58.3)	12 (50.0)	9 (47.4)	0.320
Methylxanthines, *n*(%)	3 (3.8)	2 (8)	0 (0)	0 (0)	1 (5.3)	N/A
ICS/LABA *n* (%),	29 (36.3)	9 (36.0)	4 (33.3)	8 (33.3)	8 (42.1)	0.937
ICS/LABA MART protocol, *n* (%)	30 (37.5)	11 (44.0)	4 (33.3)	9 (37.5)	6 (31.6)	0.844
Biologic, *n* (%)	17 (21.3)	7 (41.2)	2 (11.8)	6 (35.2)	2 (11.8)	N/A
Omalizumab, *n* (%)	10 (58.8)	6 (85.7)	1 (50)	2 (33.3)	1 (50)	N/A
Benralizumab, *n* (%)	7 (41.2)	1 (14.3)	1 (50)	4 (66.7)	1(50)	N/A

* For the level of significance according to chi-square test. Abbreviations: EA: eosinophilic asthma; NA: neutrophilic asthma; MGA: mixed-granulocytic asthma; PGA: paucigranulocytic asthma GINA: Global Initiative for Asthma; ACT: asthma control test; ACQ: Asthma Control Questionnaire; ICS: inhaled corticosteroid; SABA: short-acting beta 2 agonist; LAMA: long-acting muscarinic antagonists. LTRA: leukotriene receptor antagonist ICS/LABA: inhaled corticosteroid/long-acting beta 2 agonist; ICS/LABA MART: maintenance-and-reliever-therapy with ICS-formoterol; N/A: not applicable.

**Table 3:** EA, NA, MGA, and PGA markers.

Marker	Univariate Logistic Regression			Multivariate Logistic Regression		
	OR	95%CI OR	*p*	OR	95%CI OR	*p*
EA				Step 4		
Number of exacerbations during last year	1.235	1.01–1.52	0.044			
Smoke as trigger	3.857	1.24–12.04	0.020	5.966	1.53–23.30	0.010
Nasal glucocorticoids	3.054	1.06–8.81	0.039			
MEF25	0.970	0.94–0.99	0.033	0.964	0.94–0.99	0.019
MEF50	0.976	0.95–0.99	0.030			
CRP	1.198	1.01–1.43	0.049	1.246	1.01–1.54	0.043
Periostin	1.001	1.00–1.01	0.050	1.001	1.00–1.01	0.057
NA				Step 2		
Eosinophils	0.006	0.00–0.77	0.039	0.008	0.00–1.30	0.063
IL-8	1.010	1.01–1.02	0.009	1.009	1.00–1.02	0.025
IgE	0.958	0.92–0.99	0.026			
MGA				Step 4		
Allergic rhinitis in the family	2.870	1.05–7.8	0.039			
Physical activity as a trigger	7.105	1.27–39.72	0.026	5.011	0.80–31.38	0.085
Allergic immunotherapy	4.200	1.18–15.00	0.027	3.812	0.96–15.13	0.057
FEV1/FVC	1.072	1.01–1.14	0.036			
MEF50	1.027	1.01–1.05	0.021	1.030	1.01–1.06	0.023
MEF75	1.032	1.01–1.06	0.023			
PGA						
Nasal glucocorticoids	0.306	0.10–0.89	0.030			

Abbreviations: EA: eosinophilic asthma; NA: neutrophilic asthma; MGA: mixed-granulocytic asthma; PGA: paucigranulocytic asthma, ESR; erythrocyte sedimentation rate; CRP: C reactive protein; FEV1: forced expiratory volume in 1 s; FVC: forced vital capacity; MEF75: mean expiratory flow at 75% of vital capacity; MEF50: mean expiratory flow at 50% of vital capacity; MEF25: mean expiratory flow at 50% of vital capacity.

**Table 4:** Biomarkers in non-biologic patients with asthma.

Biomarker, Med (IQR)	EA *n* = 18	NA *n* = 10	MGA *n* = 18	PGA *n* = 17	*p* *
ESR (mm/h)	12 (8–22.5)	9 (7.5–18.5)	13 (9.5–18)	12 (8–17)	0.825
CRP (mg/L)	1.9 (1.5–4.2)	1.1 (1.0–2.3)	1.25 (1.0–3.4)	1.2 (1.0–3.0)	0.131
Leucocytes(10^9^/L)	6.6 (5.9–8.2)	6.4 (5.1–7.0)	6.1 (5.5–8.0)	6.0 (5.5–7.5)	0.729
Neutrophils(10^9^/L)	3.6 (2.8–4.5)	3.3 (3.0–4.6)	3.2 (2.8–4.7)	3.3 (2.6–4.0)	0.790
Lymphocytes(10^9^/L)	2.2 (1.9–2.6)	1.7 (1.2–2.6)	2.2 (1.8–2.8)	2.1 (1.7–2.2)	0.157
Eosinophils(10^9^/L)	0.3 (0.2–0.6)	0.1 (0.1–0.2)	0.2 (0.1–0.3)	0.2 (0.1–0.3)	0.034
IgE (IU/mL)	182.5 (75.7–363.5)	87.0 (60.7–288.7)	81.5 (20.2–309.5)	78.0 (18.0–205.0)	0.212
FeNO (ppb)	24.2 (14.1–39.4)	16.3 (11.6–26.3)	16.2 (7.8–32.9)	20.9 (11.6–31.6)	0.420
IL-6 (pg/mL)	2.8 (1.9–3.2)	2.4 (1.7–5.3)	2.6 (2.1–3.4)	3.1 (1.8–3.3)	0.969
Periostin (pmol/L), med	865.7 (697.6–1851.8)	661.4 (521.7–1004.1)	925.3 (713.2–1125.8)	1049.6 (836.4–1355.4)	0.256
IL-5 (pg/mL)	0.0 (0.0–0.0)	0.0 (0.0–1.0)	0.0 (0.0–0.0)	0.0 (0.0–0.0)	0.081
IL-8 (pg/mL)	0.0 (0.0–2.8)	0.2 (0.0–166.5)	0.0 (0.0–0.0)	0.0 (0.0–7.9)	0.271
IL-33 (pg/mL)	41.6 (10.8–315.0)	42.2 (3.7–406.1)	9.4 (0.2–89.7)	22.9 (4.3–194.9)	0.346
IL-17A (pg/mL)	0 (0.0–0.6)	0 (0.0–0.0)	0 (0.0–0.1)	0 (0.0–0.0)	0.004

* For the level of significance of 0.05 according to Kruskal–Wallis test. Abbreviations: EA: eosinophilic asthma; NA: neutrophilic asthma; MGA: mixed-granulocytic asthma; PGA: paucigranulocytic asthma, ESR: erythrocyte sedimentation rate; CRP: C reactive protein; FeNO: fraction of exhaled nitric oxide.

## Data Availability

The raw data supporting the conclusions of this article will be made available by the authors on request.

## Supplementary Materials

The following supporting information can be downloaded at: https://www.mdpi.com/article/10.3390/jcm13020526/s1, Table S1: Biomarkers in patients with asthma; Table S2: Biomarkers in biologic and non-biologic asthma groups.

## References

[1] Anderson G.P. (2008). Endotyping asthma: New insights into key pathogenic mechanisms in a complex, heterogeneous disease. Lancet.

[2] Wenzel S.E. (2012). Asthma phenotypes: The evolution from clinical to molecular approaches. Nat. Med..

[3] Kuruvilla M.E., Lee F.E., Lee G.B. (2019). Understanding asthma phenotypes, endotypes, and mechanism of disease. Clin. Rev. Allergy Immunol..

[4] Canonica G.W., Fernando I., Biardini F., Puggioni F., Racca F., Passalacqua G., Heffler E. (2018). Asthma: Personalized and precision medicine. Curr. Opin. Allergy Clin. Immunol..

[5] Ozdemir C., Kucukser C.U., Akdis M., Akdis C.A. (2018). The concept of asthma endotypes and phenotypes to guide current and novel treatment strategies. Expert Rev. Respir. Med..

[6] Ciprandi G., Tosca M.A., Silvestri M., Ricciardolo F.L.M. (2017). Inflammatory biomarkers in asthma endotypes and consequent personalized therapy. Expert Rev. Clin. Immunol..

[7] Kaur R., Chupp G. (2019). Phenotypes and endotypes in adult asthma: Moving toward precision medicine. J. Allergy Clin. Immunol..

[8] Gauthier M., Ray A., Wenzel S.A. (2015). Evolving Concepts of Asthma. Am. J. Respir. Crit. Care Med..

[9] Simpson J.L., Scott R., Boyle M.J., Gibson P.G. (2006). Inflammatory subtypes in asthma: Assessment and identification using induced sputum. Respirology.

[10] Feng Y., Liu X., Wang Y., Du R., Mao H. (2023). Delineating asthma according to inflammation phenotypes with a focus on paucigranulocytic asthma. Chin. Med. J..

[11] Esteban-Gorgojo I., Antolín-Amérigo D., Domínguez-Ortega J., Quirce S. (2018). Non-eosinophilic asthma: Current perspectives. J. Asthma Allergy.

[12] Svenningsen S., Nair P. (2017). Asthma endotypes and an overview of targeted therapy for asthma. Front. Med..

[13] Gibson P.G. (2009). Inflammatory phenotypes in adult asthma. Clin. Appl. Clin. Respir. J..

[14] Diamant Z., Vijverberg S., Alving K., Bakirtas A., Bjermer L., Custovic A., Dahlen S.E., Gaga M., Gerth van Wijk R., Del Giacco S. (2019). Toward clinically applicable biomarkers for asthma: An EAACI position paper. Allergy.

[15] Canonica G.W., Spanevello A., de Llano L.P., Domingo Ribas C., Blakey J.D., Garcia G., Inoue H., Dalcolmo M., Yang D., Mokashi S. (2022). Is asthma control more than just an absence of symptoms? An expert consensus statement. Respir. Med..

[16] Reddel H.K., Taylor D.R., Bateman E.D., Boulet L.P., Boushey H.A., Busse W.W., Casale T.B., Chanez P., Enright P.L., Gibson P.G. (2009). Asthma control and exacerbations: Standardizing endpoints for clinical asthma trials and clinical practice. Am. J. Respir. Crit. Care Med..

[17] Levy M.L., Bacharier L.B., Bateman E., Boulet L.P., Brightling C., Buhl R., Brusselle G., Cruz A.A., Drazen J.M., Duijts L. (2023). Key recommendations for primary care from the 2022 Global Initiative for Asthma (GINA) update. NPJ Prim. Care Respir. Med..

[18] Samitas K., Zervas E., Gaga M. (2017). T2-low asthma: Current approach to diagnosis and therapy. Curr. Opin. Pulm. Med..

[19] Fitzpatrick A.M., Chipps B.E., Holguin F., Woodruff P.G. (2020). T2-”Low” Asthma: Overview and Management Strategies. J. Allergy Clin. Immunol. Pract..

[20] Engelkes M., Janssens H.M., de Jongste J.C., Sturkenboom M.C., Verhamme K.M. (2015). Medication adherence and the risk of severe asthma exacerbations: A systematic review. Eur. Respir. J..

[21] Chung K.F., Wenzel S.E., Brozek J.L. (2014). International ERS/ATS guidelines on definition, evaluation and treatment of severe asthma. Eur. Respir. J..

[22] Nathan R.A., Sorkness C.A., Kosinski M., Schatz M., Li J.T., Marcus P., Murray J.J., Pendergraft T.B. (2004). Development of the asthma control test: A survey for assessing asthma control. J. Allergy Clin. Immunol..

[23] Juniper E.F., Bousquet J., Abetz L., Bateman E.D., GOAL Committee (2006). Identifying ‘well-controlled’ and ‘not well-controlled’ asthma using the Asthma Control Questionnaire. Respir. Med..

[24] Bernstein I.L., Li J.T., Bernstein D.I., Hamilton R., Spector S.L., Tan R., Sicherer S., Golden D.B., Khan D.A., Nicklas R.A. (2008). Allergy diagnostic testing: An updated practice parameter. Ann. Allergy Asthma Immunol..

[25] Exhaled N.O. (2005). ATS/ERS recommendations for standardized procedures for the online and offline measurement of exhaled lower respiratory nitric oxide and nasal nitric oxide. Am. J. Respir. Crit. Care Med..

[26] King G.G., Bates J., Berger K.I., Calverley P., de Melo P.L., Dellaca R.L., Farre R., Hall G.L., Ioan I., Irvin C.G. (2020). Technical standards for respiratory oscillometry. Eur. Respir. J..

[27] Djukanovic R., Sterk P.J., Fahy J.V., Hargreave F.E. (2002). Standardized methodology of sputum induction and processing. Eur. Respir. J. Suppl..

[28] Simpson J.L., McElduff P., Gibson P.G. (2010). Assessment and reproducibility of non-eosinophilic asthma using induced sputum. Respiration.

[29] Thomson N.C., Chaudhuri R., Heaney L.G., Bucknall C., Niven R.M., Brightling C.E., Menzies-Gow A.N., Mansur A.H., McSharry C. (2013). Clinical outcomes and inflammatory biomarkers in current smokers and exsmokers with severe asthma. J. Allergy Clin. Immunol..

[30] Ntontsi P., Loukides S., Bakakos P., Kostikas K., Papatheodorou G., Papathanassiou E., Hillas G., Koulouris N., Papiris S., Papaioannou A.I. (2017). Clinical, functional and inflammatory characteristics in patients with paucigranulocytic stable asthma: Comparison with different sputum phenotypes. Allergy.

[31] Crespo-Lessmann A., Curto E., Mateus Medina E.F., Palones E., Belda Soler A., Sánchez Maza S., Soto-Retes L., Plaza V. (2023). Characteristics of Induced-Sputum Inflammatory Phenotypes in Adults with Asthma: Predictors of Bronchial Eosinophilia. J. Asthma Allergy.

[32] Gao W., Han G.J., Zhu Y.J., Mao D., Hu H. (2020). Clinical characteristics and biomarkers analysis of asthma inflammatory phenotypes. Biomark. Med..

[33] Westerhof G.A., Korevaar D.A., Amelink M., de Nijs S.B., de Groot J.C., Wang J., Weersink E.J., ten Brinke A., Bossuyt P.M., Bel E.H. (2015). Biomarkers to identify sputum eosinophilia in different adult asthma phenotypes. Eur. Respir. J..

[34] Wagener A.H., De Nijs S.B., Lutter R., Sousa A.R., Weersink E.J., Bel E.H., Sterk P.J. (2015). External validation of blood eosinophils, FE(NO), and serum periostin as surrogates for sputum eosinophils in asthma. Thorax.

[35] Schleich F.N., Manise M., Sele J., Henket M., Seidel L., Louis R. (2013). Distribution of sputum cellular phenotype in a large asthma cohort: Predicting factors for eosinophilic vs neutrophilic inflammation. BMC Pulm. Med..

[36] Yap E., Chua W.M., Layaram L., Zeng I., Vandal A.C., et Garrett J. (2013). Can we predict sputum eosinophilia from clinical assessment in patients referred to an adult asthma clinic?. Intern. Med. J..

[37] Korevaar D.A., Westerhof G.A., Wang J., Cohen J.F., Spijker R., Sterk P.J., Bel E.H., Bossuyt P.M. (2015). Diagnostic accuracy of minimally invasive markers for detection of airway eosinophilia in asthma: A systematic review and meta-analysis. Lancet Respir. Med..

[38] Hastie A.T., Moore W.C., Li H., Rector B.M., Ortega V.E., Pascual R.M., Peters S.P., Meyers D.A., Bleecker E.R., Heart N. (2013). Biomarker surrogates do not accurately predict sputum eosinophil and neutrophil percentages in asthmatic subjects. J. Allergy Clin. Immunol..

[39] Comou H., Bel E.H. (2016). Improving the diagnosis of eosinophilic asthma. Expert Rev. Respir. Med..

[40] Guida G., Bagnasco D., Carriero V., Bertolini F., Ricciardolo F.L.M., Nicola S., Brussino L., Nappi E., Paoletti G., Canonica G.W. (2022). Critical evolution of asthma biomarkers in clinical practice. Front. Med..

[41] Petsky H.L., Cates C.J., Kew K.M., Chang A.B. (2018). Tailoring asthma treatment on eosinophilic markers (exhaled nitric oxide or sputum eosinophils): A systematic review and meta-analysis. Thorax.

[42] Dweik R.A., Boggs P.B., Erzurum S.C., Irvin C.G., Leigh M.W., Lundberg J.O., Olin A.C., Plummer A.L., Taylor D.R., American Thoracic Society Committee on Interpretation of Exhaled Nitric Oxide Levels (FENO) for Clinical Applications (2011). An Official ATS Clinical Practice Guideline: Interpretation of Exhaled Nitric Oxide Levels (FENO) for Clinical Applications. Am. J. Respir. Crit. Care Med..

[43] Cottini M., Bondi B., Bagnasco D., Braido F., Passalacqua G., Licini A., Lombardi C., Berti A., Comberiati P., Landi M. (2023). Impulse oscillometry defined small airway dysfunction in asthmatic patients with normal spirometry: Prevalence, clinical associations, and impact on asthma control. Respir. Med..

[44] Marseglia G.L., Cirillo I., Vizzaccaro A., Klersy C., Tosca M.A., La Rosa M., Marseglia A., Licari A., Leone M., Ciprandi G. (2007). Role of forced expiratory flow at 25–75% as an early marker of small airway impairment in subjects with allergic rhinitis. Allergy Asthma Proc..

[45] Cirillo I., Klersy C., Marseglia G.L., Vizzaccaro A., Pallestrini E., Tosca M., Ciprandi G. (2006). Role of FEF 25–75% as a predictor of bronchial hyperreactivity in allergic patients. Ann. Allergy Asthma Immunol..

[46] Postma D.S., Brightling C., Baldi S., Van den Berge M., Fabbri L.M., Gagnatelli A., Papi A., Van der Molen T., Rabe K.F., Siddiqui S. (2019). Exploring the relevance and extent of small airways dysfunction in asthma (ATLANTIS): Baseline data from a prospective cohort study. Lancet Respir. Med..

[47] Cottini M., Lombardi C., Passalacqua G., Bagnasco D., Berti A., Comberiati P., Imeri G., Landi M., Heffler E. (2022). Small Airways: The “Silent Zone” of 2021 GINA Report?. Front. Med..

[48] Abdo M., Pedersen F., Kirsten A.M., Veith V., Biller H., Trinkmann F., von Mutius E., Kopp M., Hansen G., Rabe K.F. (2022). Longitudinal Impact of Sputum Inflammatory Phenotypes on Small Airway Dysfunction and Disease Outcomes in Asthma. J. Allergy Clin. Immunol. Pract..

[49] Lu L., Peng J., Zhao N., Wu F., Tian H., Yang H., Deng Z., Wang Z., Xiao S., Wen X. (2022). Discordant Spirometry and Impulse Oscillometry Assessments in the Diagnosis of Small Airway Dysfunction. Front. Physiol..

[50] Liwsrisakun C., Chaiwong W., Pothirat C. (2023). Comparative assessment of small airway dysfunction by impulse oscillometry and spirometry in chronic obstructive pulmonary disease and asthma with and without fixed airflow obstruction. Front. Med..

[51] Ciółkowski J., Emeryk A., Hydzik P., Emeryk-Maksymiuk J., Kosmala E., Stasiowska B. (2019). Eosinophilic airway inflammation is a main feature of unstable asthma in adolescents. Respir. Med..

[52] Jia G., Erickson R.W., Choy D.F., Mosesova S., Wu L.C., Solberg O.D., Shikotra A., Carter R., Audusseau S., Hamid Q. (2012). Periostin is a systemic biomarker of eosinophilic airway inflammation in asthmatic patients. J. Allergy Clin. Immunol..

[53] Simpson J.L., Yang I.A., Upham J.W., Reynolds P.N., Hodge S., James A.L., Jenkins C., Peters M.J., Jia G., Holweg C.T. (2016). Periostin levels and eosinophilic inflammation in poorly controlled asthma. BMC Pulm. Med..

[54] Shimoda T., Obase Y., Kishikawa R., Iwanaga T. (2015). Serum high-sensitivity C-reactive protein can be an airway inflammation predictor in bronchial asthma. Allergy Asthma Proc..

[55] Ali H., Douwes J., Burmanje J., Gokhale P., Crane J., Pattemore P., Stanley T., Keenan J., Brooks C. (2023). Sputum inflammatory, neural, and remodeling mediators in eosinophilic and noneosinophilic asthma. Ann. Allergy Asthma Immunol..

[56] Wood L.G., Baines K.J., Fu J., Scott H.A., Gibson P.G. (2012). The neutrophilic inflammatory phenotype is associated with systemic inflammation in asthma. Chest.

[57] Lambrecht B.N., Hammad H. (2021). The basic immunology of asthma. Cell.

[58] Bullone M., Carriero V., Bertolini F., Folino A., Mannelli A., Di Stefano A., Gnemmi I., Torchio R., Ricciardolo F.L. (2019). Elevated serum IgE, oral corticosteroid dependence, and IL-17/22 expression in highly neutrophilic asthma. Eur. Respir. J..

[59] Gibson P.G., Foster P.S. (2019). Neutrophilic asthma: Welcome back!. Eur. Respir. J..

[60] Demarche S., Schleich F., Henket M., Paulus V., Van Hees T., Louis R. (2016). Detailed analysis of sputum and systemic inflammation in asthma phenotypes: Are paucigranulocytic asthmatics really non-inflammatory?. BMC Pulm. Med..

[61] Dimitrova D., Youroukova V., Ivanova-Todorova E., Tumangelova-Yuzeir K., Velikova T. (2019). Serum levels of IL-5, IL-6, IL-8, IL-13 and IL-17A in pre-defined groups of adult patients with moderate and severe bronchial asthma. Respir. Med..

[62] Luo W., Hu J., Xu W., Dong J. (2022). Distinct spatial and temporal roles for Th1, Th2, and Th17 cells in asthma. Front. Immunol..

[63] Solidoro P., Nicola S., Ridolfi I., Canonica G.W., Blasi F., Paggiaro P., Heffler E., Bagnasco D., Patrucco F., Ribolla F. (2022). Biologics in Severe Eosinophilic Asthma: Three-Year Follow-Up in a SANI Single Center. Biomedicines.

[64] Šokić M.K., Rijavec M., Korošec P., Bidovec-Stojkovič U., Kern I., Vantur R., Škrgat S. (2022). Heterogeneous Response of Airway Eosinophilia to Anti-IL-5 Biologics in Severe Asthma Patients. J. Pers. Med..

[65] Cosmi L., Maggi L., Santarlasci V., Capone M., Cardilicchia E., Frosali F., Querci V., Angeli R., Matucci A., Fambrini M. (2010). Identification of a novel subset of human circulating memory CD4(+) T cells that produce both IL-17A and IL-4. J. Allergy Clin. Immunol..

[66] Irvin C., Zafar I., Good J., Rollins D., Christianson C., Gorska M.M., Martin R.J., Alam R. (2014). Increased frequency of dual-positiveTH2/TH17 cells in bronchoalveolar lavage fluid characterizes a pop-ulation of patients with severe asthma. J. Allergy Clin. Immunol..

[67] Wakashin H., Hirose K., Maezawa Y., Kagami S.I., Suto A., Watanabe N., Saito Y., Hatano M., Tokuhisa T., Iwakura Y. (2008). IL-23 and Th17 cells enhance Th2-cell-mediated eosinophilic airway inflammation in mice. Am. J. Respir. Crit. Care Med..

[68] Hasegawa T., Uga H., Mori A., Kurata H. (2017). Increased serum IL-17A and Th2 cytokine levels in patients with severe uncontrolled asthma. Eur. Cytokine Netw..

[69] Wang Y.H., Voo K.S., Liu B., Chen C.Y., Uygungil B., Spoede W., Bernstein J.A., Huston D.P., Liu Y.J. (2010). A novel subset of CD4(+) T(H)2memory/effector cells that produce inflammatory IL-17 cytokine and promote the exacerbation of chronic allergic asthma. J. Exp. Med..

[70] Pouliquen I.J., Kornmann O., Barton S.V., Price J.A., Ortega H.G. (2015). Characterization of the relationship between dose and blood eosinophil response following subcutaneous administration of mepolizumab. Int. J. Clin. Pharmacol. Ther..

[71] Tsukamoto N., Takahashi N., Itoh H., Pouliquen I. (2016). Pharmacokinetics and pharmacodynamics of mepolizumab, an anti-interleukin 5 monoclonal antibody, in healthy Japanese male subjects. Clin. Pharmacol. Drug Dev..

[72] Pham T.H., Damera G., Newbold P., Ranade K. (2016). Reductions in eosinophil biomarkers by benralizumab in patients with asthma. Respir. Med..

[73] Takaku Y., Soma T., Nishihara F., Nakagome K., Kobayashi T., Hagiwara K., Kanazawa M., Nagata M. (2013). Omalizumab attenuates airway inflammation and interleukin-5 production by mononuclear cells in patients with severe allergic asthma. Int. Arch. Allergy Immunol..

[74] Caminati M., Marcon A., Guarnieri G., Miotti J., Bagnasco D., Carpagnano G.E., Pelaia G., Vaia R., Maule M., Vianello A. (2023). Benralizumab Efficacy in Late Non-Responders to Mepolizumab and Variables Associated with Occurrence of Switching: A Real-Word Perspective. J. Clin. Med..

[75] Van Veen I.H., Ten Brinke A., Gauw S.A., Sterk P.J., Rabe K.F., Bel E.H. (2009). Consistency of sputum eosinophilia in difficult-to-treat asthma: A 5-year follow-up study. J. Allergy Clin. Immunol..

[76] Green R.H., Pavord I. (2012). Stability of inflammatory phenotypes in asthma. Thorax.

[77] Hancox R.J., Cowan D.C., Aldridge R.E., Cowan J.O., Palmay R., Williamson A., Town G.I., Taylor D.R. (2012). Asthma phenotypes: Consistency of classification using induced sputum. Respirology.

[78] Betancor D., Olaguibel J.M., Rodrigo-Muñoz J.M., Arismendi E., Barranco P., Barroso B., Bobolea I., Cárdaba B., Cruz M.J., Curto E. (2022). How reliably can algorithms identify eosinophilic asthma phenotypes using non-invasive biomarkers?. Clin. Transl. Allergy.

