# Supplementary material for: Asthma Inflammatory Phenotypes: How Can We Distinguish Them?
Source: J Clin Med. 2024 Jan 17;13(2):526. doi: 10.3390/jcm13020526 (PMC10816410; doi:10.3390/jcm13020526)
Supplement: Supplementary file 1 [file jcm-13-00526-s001.zip › jcm-2765126-supplementary.pdf]

Table S1. Biomarkers in asthma patients.

Biomarker, med (IQR)	Total	EA n=25	NA n=12	MGA n=24	PGA n=19	p*
ESR (mm/h)	12.0 (8.0-18.0)	12.0 (9.0-22.0)	11.0 (8.0-28.0)	13.0 (10.0-19.0)	12.0 (8.0-16.0)	0.967
CRP (mg/L)	1.5 (1.0-3.2)	1.9 (1.4-3.6)	1.0 (1.0-1.9)	1.5 (1.0-4.1)	1.2 (1.0-2.9)	0.070
Leucocytes(10^9/L)	6.3 (5.5-7.3)	6.5 (6.0-7.8)	6.4 (5.1-7.2)	5.7 (5.3-6.8)	6.3 (5.6-7.2)	0.315
Neutrophils(10^9/L)	3.3 (2.8-4.2)	3.5 (3.0-4.1)	3.3 (3.1-4.7)	2.9 (2.6-3.6)	3.3 (2.4-4.5)	0.209
Lymphocytes(10^9/L)	2.1 (1.7-2.5)	2.2 (1.9-2.6)	1.7 (1.2-2.6)	2.1 (1.7-2.6)	2.1 (1.7-2.3)	0.328
Eosinophils(10^9/L)	0.2 (0.1-0.4)	0.4 (0.2-0.6)	0.1 (0.02-0.2)	0.1 (0.1-0.3)	0.2 (0.1-0.3)	**0.002**
IgE (IU/ml)	121.5 (56.2-364.5)	186.0 (75.5-414.5)	105.0 (63.0-274.2)	116.0 (37.2-353.2)	78.0 (18.0-270.0)	0.224
FeNO (ppb)	23.5 (12.1-37.7)	26.6 (15.0-48.0)	21.3 (11.9-31.8)	20 (8.6-37.5)	22.1 (12.0-35.0)	0.349
IL-6 (pg/ml)	2.7 (2.0-3.3)	2.6 (1.6-3.3)	2.5 (1.8-3.0)	2.8 (2.1-3.5)	2.8 (2.1-3.2)	0.747
Periostin (mmol/l), med	924.4 (697.5-1325.9)	950.8 (747.3-1819.5)	661.4 (517.4-1038.8)	907.8 (699.2-1212.1)	1049.6 (858.4-1397.6)	0.116
IL-5 (pg/ml)	0.0 (0.0-0.0)	0.0 (0.0-0.3)	0.0 (0.0-1.8)	0.0 (0.0-0.0)	0.0 (0.0-0.0)	0.133
IL-8 (pg/ml)	0.0 (0.0-11.5)	0.0 (0.0-5.8)	0.5 (0.0-173.8)	0.0 (0.0-7.4)	0.0 (0.0-15.9)	0.200
IL-33 (pg/ml)	21.0 (3.8-122.1)	28.6 (4.9-145.7)	31.9 (6.6-250.8)	16.8 (0.5-78.9)	22.9 (8.7-138.1)	0.703
IL-17A (pg/ml)	0.0 (0.0-0.0)	0.0 (0.0-0.3)	0.0 (0.0-0.0)	0.0 (0.0-0.2)	0.0 (0.0-0.0)	0.115

* For the level of significance of 0.05 according to Kruskal-Wallis test.

Abbreviations:EA:eosinophilic asthma; NA:neutrophilic asthma; MGA:mixed-granulocytic asthma;PGA: paucigranulocytic asthma, ESR: erythrocyte sedimentation rate; CRP:C reactive protein; FeNO: fraction of exhaled nitric oxide.

Table S2. Biomarkers in biologic and non-biologic asthma group.

Biomarker, med (IQR)	EA Bio n=7	EA No bio n=18	p*	NA Bio n=2	NA No bio n=10	p*	MGA Bio n=6	MGA No bio n=18	p*	PGA Bio n=2	PGA No bio n=17	p*
ESR (mm/h)	12 (10-24.0)	12 (8-22.5)	0.879	Const	9(2-36)	N/A	13.5 (8.0-24.0)	13.0 (9.5-18.0)	0.712	9.0 (6.0-9.0)	12.0 (8.0-17.0)	0.348
CRP (mg/L)	1.9 (1.0-2.5)	1.9 (1.5-4.2)	0.831	Const	1.1 (1-7.1(	N/A	4.6 (1.4-7.5)	1.2 (1.0-3.4)	0.062	1.1 (1.0-1.1)	1.2 (1.0-3.0)	0.584
Leu(10^9/L)	6.5 (6.3-7.7)	6.6 (5.9-8.2)	0.808	8.4 (5.1-8.4)	6.4 (5.1-7.0)	0.666	5.3 (4.6-5.8)	6.1 (5.5-8.0)	**0.049**	7.15 (7.1-7.15)	6.0 (5.5-7.5)	0.424
Ne10^9/L)	3.1 (3.0-3.5)	3.6 (2.8-4.5)	0.524	5.6 (3.4-5.6)	3.3 (3.0-4.6)	0.194	2.6 (2.1-2.7)	3.2 (2.8-4.7)	**0.019**	3.5 (2.3-3.5)	3.3 (2.6-4.0)	0.894
Ly(10^9/L)	2.2 (1.1-2.7)	2.2 (1.9-2.6)	0.745	2.1 (1.2-2.1)	1.7 (1.2-2.6)	0.747	2.0 (1.6-2.3)	2.2 (1.8-2.8)	0.270	2.6 (1.4-2.6)	2.1 (1.7-2.2)	0.788
Eo(10^9/L)	0.4 (0.2-0.9)	0.35 (0.2-0.6)	0.583	Const	0.15(0-0.3)	N/A	0.05 (0.0-0.2)	0.2 (0.1-0.3)	0.068	2.0 (0.1-2.0)	0.2 (0.1-0.3)	0.543
IgE (IU/ml)	370 (38.0-654.0)	182.5 (75.7-363.5)	0.545	245(53-730)	87(53-730)	0.283	150.5 (108.7-524.7)	81.5 (20.2-309.5)	0.161	444.5 (39.0-444.5)	78.0 (18.0-205.0)	0.425

FeNO (ppb)	46.6 (24.1-84.1)	24.20 (14.1-39.4)	0.069	51.6 (33.0-51.6)	16.3 (11.6-26.3)	0.053	62.9 (8.7-62.9)	16.2 (7.8-16.2)	0.102	79.0 (35.0-79.0)	20.9 (11.6-31.6)	0.063
IL-6(pg/ml)	1.7 (1.5-3.5)	2.8 (1.9-3.2)	0.190	Const	2.45(1.6-14.2)	N/A	3.3 (3.1-4.9)	2.6 (2.1-3.4)	**0.032**	2.4 (2.1-2.4)	3.1 (1.8-3.3)	0.594
Periostin (mmol/l), med	1330.1 (783.1-1837.2)	865.7 (697.6-1851.8)	0.333	795.8 (514.7-795.8)	661.4 (521.7-661.4)	0.830	823.8 (611.5-1807.7)	925.3 (713.2-1125.8)	0.841	1506.8 (995.5-1506.8)	1049 (836.4-1355.4)	0.352
IL-5 (pg/ml)	0.3 (0.0-1.1)	0.0 (0.0-0.0)	**0.043**	4.1 (0.4-4.1)	0.0 (0.0-1.0)	0.108	0.04 (0.0-4.1)	0.0 (0.0-0.0)	**0.012**	6.9 (0.0-6.9)	0.0 (0.0-0.0)	0.117
IL-8 (pg/ml)	0.0 (0.0-13.6)	0.0 (0.0-2.8)	0.422	160.1 (16.1-160.1)	0.2 (0.0-166.6)	0.181	6.1 (0.0-33.0)	0.0 (0.0-0.0)	**0.038**	11.9 (0.0-11.9)	0.0 (0.0-7.9)	0.549
IL-33 (pg/ml)	3.8 (1.8-100.9)	41.6 (10.8-315.0)	0.130	15.3 (11.8-15.3)	45.2 (3.7-406.1)	0.667	48.5 (3.0-209.7)	9.4 (0.2-89.7)	0.316	48.6 (16.4-48.6)	22.9 (4.3-194.9)	0.790
IL-17A (pg/ml)	0.0 (0.0-0.5)	0.0 (0.0-1.2)	0.339	2.45(2-2.9)	Const	N/A	0.0 (0.0-1.2)	0.0 (0.0-0.2)	0.661	4.3 (0-8.7)	Const	N/A

* For the level of significance of 0.05 according Mann- Whitney test.

Abbreviations:EA:eosinophilic asthma; NA:neutrophilic asthma; MGA:mixed-granulocytic asthma;PGA: paucigranulocytic asthma; Bio; biologis, No bio: no biologics; ESR: erythrocyte sedimentation rate; CRP:C reactive protein; Le: leucocytes; Ne: neutrophils; Ly: lymphocyte; Eo: eosinophils; FeNO: fraction of exhaled nitric oxide, Const: constant value with variability equals; NA: not applicable.
